# Use of TIRF to Monitor T-Lymphocyte Membrane Dynamics with Submicrometer and Subsecond Resolution

**DOI:** 10.1007/s12195-014-0361-8

**Published:** 2014-10-22

**Authors:** Alexandre Brodovitch, Laurent Limozin, Pierre Bongrand, Anne Pierres

**Affiliations:** 1 Laboratoire Adhésion et Inflammation, Parc Scientifique de Luminy, Aix-Marseille Université, INSERM U1067, Case 937, 13288 Marseille Cedex 09, France; 2 INSERM U 1067, 13288 Marseille Cedex 09, France; 3 CNRS U 7333, 13288 Marseille Cedex 09, France; 4 Assistance-Publique, Hôpitaux de Marseille, Marseille, France

**Keywords:** T-lymphocyte, Antigen detection, Microvilli, Membrane movement, Interface, Total internal reflection fluorescence

## Abstract

A key step of adaptive immune responses is the T lymphocyte capacity to detect the presence of foreign antigens on specialized cells with high speed and specificity during contacts lasting a few minutes. Much evidence suggests that there is a deep link between the lifetime of molecular interactions between T cell receptors and ligands and T cell activation, but the precise mechanisms of bond formation and dissociation remain incompletely understood. Previous experiments done with interference reflection microscopy/reflection interference contrast microscopy disclosed transverse motions with several nanometer average amplitude of micrometer size membrane zones. More recently, total internal reflection fluorescence microscopy was used to show that the initial interaction between primary T lymphocytes and model surfaces involved the tip of microvilli (typically 0.2 *µ*m^2^ area) generating apparent contacts of a few seconds that allowed cells to detect ligands of their membrane receptors. Here we show that these microvilli displayed minimal lateral displacements but quantitative fluorescence measurement suggested the occurrence of spontaneous transverse fluctuations of order of 67 nm amplitude during 1-s observation periods. This may play a major role in membrane receptor engagement and ensuing signal generation.

## Introduction

### T Lymphocyte Function

A major task of T lymphocytes consists of patrolling throughout living organisms to scan surrounding cells in order to detect the presence of foreign material consisting of oligopeptides (*p*) bound to molecules encoded by the major histocompatibility complex (MHC).^4^ An antigen presenting cell (APC) may express 100,000 MHC molecules exposing thousands of different peptide species. Therefore, only a few tens or less of pMHC complexes specific for a given protein molecule may appear on the cell membrane. A T lymphocyte bears a single antigen receptor (TCR) species on its membrane. This must allow accurate discrimination between pMHCs that may differ by a single aminoacid.^10^ Much previous evidence supported the hypothesis that this discrimination should be based on quantitative binding parameters rather than geometrical features.^11^ An early finding was that the capacity of a pMHC to activate a T cell is positively correlated to the lifetime of TCR/pMHC interaction.^25^ However, until recently, only so-called 3D interactions involving at least one free ligand species had been studied quantitatively, and it was well understood that available data could not account for so-called 2D interactions between membrane-bound TCRs and pMHCs. During the last years, a few authors reported on interactions between surface-bound TCRs and pMHCs. A laminar flow chamber was used to study bond formation between a TCR and 8 pMHC species bound to artificial surfaces.^32^ 2D dissociation rates were well correlated to 3D parameters previously measured on the same molecules.^2^ In contrast, experiments made on different molecular systems involving living T cells suggested that 2D dissociation rates were much higher than 3D dissociation rates, and this difference was abolished by cytoskeletal inhibitors.^19^ In another study, 2D dissociation rates appeared positively correlated to activation efficiencies, in contrast with 3D dissociation rates.^18^ More recent evidence suggested that the capacity of TCR-pMHC interaction to activate T cells might be related to their capacity to be stabilized by external forces, a property specific of so-called *catch bonds*.^23^ Other authors reported strong evidence that the TCR might act as a mechanotransducer.^20^,^22^ Thus, membrane dynamics at the T-lymphocyte/APC interface might strongly influence TCR-pMHC interaction under physiological conditions, either through force application and mechanotransduction or through modulation of interaction lifetimes.^14^

Therefore, there is a strong interest in obtaining quantitative information on the movements of cell membranes with a time resolution within the second range, corresponding to the typical lifetime of TCR/pMHC interaction, and a distance resolution close to the length of the TCR/pMHC couple, which is about 15 nm.

Recently, two microscopical techniques were found to yield this kind of information: interference reflection microscopy (IRM), also called reflection interference contrast microscopy (RICM), and total internal reflection fluorescence microscopy (TIRFM).

### Studying T Lymphocyte Encounter with Activating and Non-Activation Surfaces with IRM

T lymphocytes were made to sediment on surfaces coated with (non-activating) anti-HLA or (activating) anti-CD3 monoclonal antibodies (CD3 is tightly associated with the TCR). When cells were examined with quantitative IRM, It was found^7^,^8^ that cells falling on glass surfaces first appeared as localized spots on fluctuation maps. Fluctuation amplitude was typically on the order of 10 nm. Further, after a lag of about 1 min, cells began to spread. In accordance with previous reports,^6^ the spreading velocity was much higher on activating surfaces than on non-activating surfaces, demonstrating that a first phase of antigen detection was completed within 1 min.

However, intrinsic limitation of IRM lateral resolution made it impossible to obtain accurate information on the shape of cell membrane regions involved in initial contact with surfaces. This was an incentive to use TIRFM in order to obtain more accurate information on this key step of T lymphocyte activation.

### Studying Lymphocyte Membrane Dynamics at Interfaces with TIRF

Evanescent wave illumination was used by Axelrod^3^ to image selectively cell-substrate contacts. The basic principle consists of illuminating a glass-sample interface with a light ray incidence higher than the critical angle θ_c_ = Arcsin (*n*_1_/*n*_2_), where *n*_1_ and *n*_2_ are respectively the refractive index in the sample and the glass coverslip. This generates an evanescent wave the intensity of which displays exponential decay with respect to the distance *z* to the interface:

1 $$ I = I_{0} \exp \left( { - z/d} \right) $$

2 $$ d = (\lambda_{0} /4\pi )\left( {n_{2}^{2} \sin^{2} \theta {-}n_{1}^{2} } \right)^{ - 1/2} $$

where *I* is the local intensity, θ is the light incidence and λ_0_ is the light wavelength in vacuum.^26^,^27^ The penetration depth d is typically 100–200 nm. Suitable incidences were first achieved by gluing a quartz or a glass block on the coverslip. More recently, commercial equipments were made available with through-the-objective illumination.^26^ While it may appear tempting to use Eqs. (1) and (2) to derive sample-to-interface distance from image brightness, some points of caution must be emphasized: (i) the actual incidence angle may be difficult to determine accurately, (ii) as for IRM, the presence in the sample of several layers of variable refractive index affects the measured signal, (iii) the illumination field may not be rigorously exponential due to reflections in the objective,^26^ (iv) if the microscope is focused on the glass-medium interface, the image of an out-of-focus fluorescent dot on the sample is dependent on the defocusing distance,^13^ and (v) it must be ensured that the observed sample is uniformly labeled and that no fluorophore bleaching occurs during the observation period.

**Figure 1:**
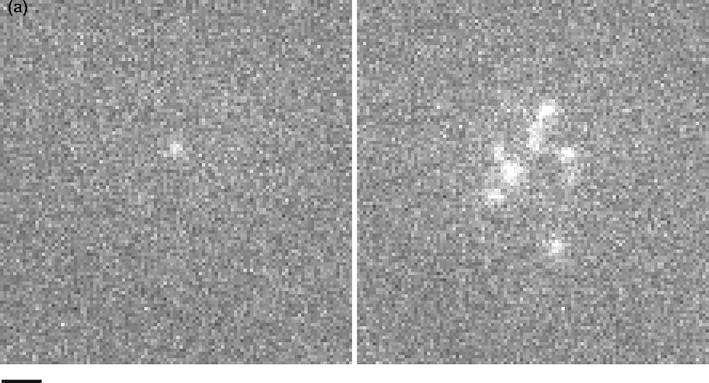
Using TIRFM to image cell membrane at interfaces. A T lymphocyte falling on a neutral (non-adhesive, non-activting surfaces) after fluorescent membrane labeling was monitored with TIRFM. Two sequential images of a same cell separated by a 20 s time period are shown. A single spot is visible on the left. Bar length = 1 *µ*m

As a consequence, apart from notable exceptions^1^,^16^,^17^,^28^ most authors used TIRFM in a qualitative way to image cell-substrate contacts. We used this approach to study the initial contact between membrane-labeled primary T lymphocytes and activating or non-activating surfaces and monitoring. The following conclusions were obtained^5^: First, contrast was much better with TIRFM than with IRM. The cell membrane region in close proximity to the underlying surface was brighter than the background by more than 12× standard deviation. It was thus easily visualized by thresholding without any filtering. Further, during the first minute following cell-to-surface encounter, cell images were made of transient contact spots of typically 0.4 *µ*m diameter and a lifetime ranging from less than a second to a minute or more (Fig. 1). Spreading began after 30–40 contacts formed during a lag of about 1 min duration. Based on electron microscopic evidence,^33^ a reasonable interpretation would be that these bright spots represented the tip of microvilli.

The purpose of the present paper was to use quantitative processing of TIRFM images to derive additional information on the dynamics of the tip of T-lymphocyte microvilli during cell-substratum contact. It is concluded that molecular contacts between cells and surfaces are mediated by highly dynamic microvilli of submicrometer diameter undergoing spontaneous movements with a typical duration of a few seconds or less and amplitude lower than 100 nm normal to the surface. These results support the possibility that living cells might probe their environment with a timescale much shorter than was previously estimated. It is suggested that this approach might allow a better understanding of TCR-mediated T-lymphocyte activation^14^ and provide new ways of assessing T lymphocyte function in hospital practice.^8^

## Materials and Methods

### Cells

In order to optimize the physiological relevance of our results, we used outdated peripheral blood from healthy volunteers, as supplied by the French blood bank. These volunteers had been informed that blood might be used for research purpose, in accordance with the ethical policy of our institution. Peripheral blood mononuclear cells were purified by density gradient centrifugation following standard procedures. CD4+ T lymphocytes were then isolated by negative selection with magnetic cell sorting (Miltenyi Biotec), in order to prevent any undesirable activation.^5^,^8^

Fluorescent labeling of plasma membrane for TIRFM was obtained by incubating cells with 15 *µ*g/mL 4-(4-(dihexadecylamino)styryl)-*N*-methylpyridinium iodide (DiA; Molecular Probes, Invitrogen) for 40 min at 37°^5^ In some experiments, cells fixation was obtained by 10 min incubation with 3.7% paraformaldehyde (Sigma).^29^

### Surfaces

Glass coverslips were cleaned with sulphuric acid before sequential treatment with (3-aminopropyl)triethoxysilane, then 1% glutaraldehyde, then antibodies. Reactive groups were blocked with ethanolamine and coverslips were thoroughly washed. Antibodies were IgG1 controls (in order to minimize adhesive interactions) as provided by Beckmann-Coulter.^5^

### Total Internal Reflection Microscopy

We used an inverted Axiovert 200 M microscope (Zeiss) bearing a polarized laser (series 77, LASOS lasertechnik, Jena), a 100× objective (1.45 NA, 110 *µ*m working distance) and a filter cube with 458/10 excitation, 470 dichroic and 605/40 emission filters. The refractive index of immersion oil was 1.518. Images were captured with an iXon camera (Andor, Belfast). Pixel size was 80 × 80 nm^2^. Exposure time was 200 ms. Frame rate was 4.90 Hz.^5^ During microscopic monitoring, cells were maintained at 37°C in a heating chamber.

### Image Processing

Images were processed with a custom-made software written in C++^30^ or with image J (National Institutes of Health, Wayne Rasband; http://rsb.info.nih.gov/ij) supplemented with custom-made plugins. The following points were found important: Early cell-to-surface contacts were easily detected on TIRFM images where they appeared as bright spots of typically 5 pixel linear size. However, it was desirable to define the boundaries of these spots with maximum accuracy in order to analyze lateral and vertical displacements. This was achieved by means of a contour-follow algorithm that has been used for decades in our laboratory. This consisted of defining the spot frontier as the boundary between pixels with a brightness respectively higher and lower than a chosen threshold value v_t_. The coordinates of the image centroid or center of gravity could then be calculated with subpixel accuracy. The specific spot brightness was then calculated by background subtraction. The most convenient way of determining the background was found to use the *mode*, i.e., the most frequent pixel brightness on a full image of 1002 × 1004 pixels. The temporal autocorrelation function as defined by Gaborski *et al*.^12^ was calculated with a custom-made ImageJ plugin (source is available on request). Optimal results were obtained by first using mean filtering (as implemented in ImageJ, *r* = 2 pixels) in order to minimize the effect of random brightness fluctuations. Simulated images of spheres and membrane protrusions were built with a custom-made Java program based on standard finite element method. The illumination intensity of a defocused point was estimated with mono- or bi-exponential formulae.^26^ The image of a defocused point was obtained with a theoretical estimate of the point spread function^13^ accounting for the objective working distance and oil refraction index, as implemented by PSF-generator ImageJ plugin built by Kirshner and Sage,^21^ downloadable at EPFL website (http://bigwww.epfl.ch/algorithms/), assuming 100% collection efficiency of emission light.^15^

## Results and Discussion

### Using TIRF to Look for Lateral and Normal Motion of Lymphocyte Surface Microvilli

While the boundaries of well-spread cells were immediately apparent on TIRF images,^5^ analysis of transient contacts was much more demanding since this required high temporal resolution and contrast amplification (Fig. 1). It was therefore important to examine the quality of images to determine the ultimate performance that might be expected.

#### Noise Analysis

First, we examined the noise on a series of 1042 images recorded with a frame rate of 4.9 Hz. We chose a 49 pixel area that remained empty throughout the observation period. The average brigthness was 1019.1 ± 2.3 Standard Deviation. A representative pixel within this area yielded 1019.2 ± 8.2 SD. The pixel brightness was only weakly correlated to the average area brightness (correlation coefficient *r* = 0.20), and the coefficient of variation of individual pixel brightness (CV = 8.2/1019 = 0.008) was not improved by dividing pixel intensity with the average field brightness (yielding CV = 0.0079). Also, the SD of individual pixel brightness was not reduced by subtracting the average brightness of the image. Thus, no average brightness correction was performed to compensate for local fluctuations of pixel brightness.

**Figure 2:**
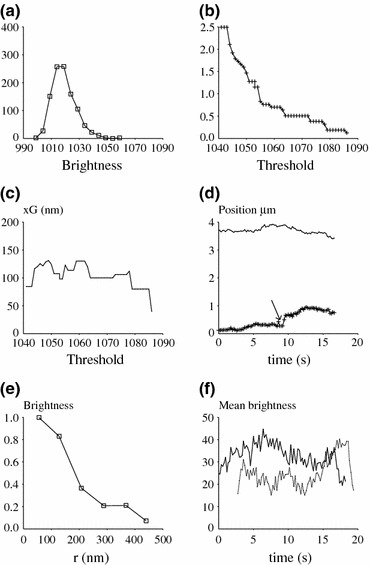
Quantitative processing of cell images. (a) Fluctuations of single pixel brightness. 1042 sequential images were used to determine the brightness fluctuations of a single pixel located in a cell-free area. The frequency distribution is shown. This appears as a skewed gaussian with enlarged rightward tail. (b) Dependence of calculated spot area on threshold value. The area of a representative fluorescent spot was determined with a computerized algorithm based on an arbitrary threshold value. As shown on the figure, the calculated area is highly dependent on the threshold (background intensity is 1021) and this cannot be used as a robust reporter of spot size. (c) Dependence of calculated spot position on threshold value. In contrast with the calculated area (Fig. 2b), the position of the centroid of a fluorescent spot is only weakly dependent on the threshold value. (d) In-plane displacement of cell surface microvilli. The positions of the tips of two microvilli displayed by a same cell were recorded with 4.9 Hz frequency. The variation of centroid abscissa are shown as two separate curves: spots displayed rare shifts of a few tenths of a micrometer (arrow). (e) Radial distribution of spot brightness. The radial fluorescence distribution of a representative spot is shown. (f) Brightness fluctuations of spot images. The fluorescence of microvilli tips was estimated as fluorescence of discs of 320 nm radius with background subtraction. The fluorescence of two representative spots displayed by a same cell is shown (full and dotted lines). Images are suggestive of independent displacements with 5–10 s duration

The brightness distribution at individual pixels was also studied: As shown on Fig. 2a, this was skewed with a larger tail than a gaussian curve. Nearly 5% and 1% of values were respectively higher than the mean by 15 and 24 fluorescence units, i.e., about 2 and 3 SD. This is twice as high as values yielded by the normal distribution law.

These results provided guidelines for choosing threshold values in order to determine the boundaries of cell membrane protrusions approaching surfaces.

#### Studying Lateral Displacements of Cell Contacts

As shown on Fig. 1, cell protrusion tips approaching a surface appeared as small clusters of pixels with a brightness ranging between typically 1030 and 1080 units as compared to a background of 1020. It was not felt warranted to locate these spots by mathematical fitting with theoretical distributions in view of the limited number of pixels and unknown reference shape: indeed, it is not ensured that contact spots had a circular shape, and contact spot fluorescence might be superimposed on contributions of the cell body. Therefore, we used two simpler procedures that consisted either of building the spot boundary by starting with a “reasonable” threshold or determining the center of a sliding disc corresponding to maximum brightness. The following conclusions were obtained: First, the calculated spot area was highly dependent on the threshold value: as shown in a representative example (Fig. 2b), the calculated spot area exhibited nearly fivefold decrease when the threshold was varied within a seemingly reasonable range from 1040 to 1060. Second, the spot position defined as the centroid G of the thresholded contour was very weakly dependent on the threshold: in a representative example depicted on Fig. 2c, the SD of G abscissa was only 15 nm when the threshold intensity was varied between 1040 and 1080. In contrast, the delimited area decreased from 39 to 3 pixels. Third, very similar results were obtained when the spot position was obtained by maximizing the total brightness of a disc with a radius varying up to 7 pixels.

It was concluded that the thresholding algorithm we used provided a robust way of determining the position of cell protrusions tip with subpixel accuracy. This was used to follow a set of ten representative spots (469 sequential positions). Coordinates *x* and *y* were calculated. The standard deviation of *x* and *y* coordinates ranged between 46 and 348 nm (mean 131 nm) on the 20 sets of coordinates that were studied. More precise information was obtained by examining individual trajectories. The *y* position of two spots followed on a same cell during a time interval of 15 s is shown on Fig. 2d. It was concluded that the lateral displacements of protrusion tips remained low. Periods of undetectable displacement (less than about 100 nm amplitude, which is close to the accuracy of position determination), were interspersed with rapid displacements of a few tenths of a micrometer (Fig. 2d, arrow). Also, contact spots formed by a same cell might display independent lateral displacements (Fig. 2d).

*In conclusion*, cell contact with nonadhesive surfaces displayed only limited lateral movements.

#### Studying Vertical Displacements of Cell Protrusions

It was warranted to ask whether cell surface protrusions displayed vertical oscillations with a period on the order of seconds. While single TIRFM images such as are displayed in Fig. 1 did not allow us to reconstruct the full membrane shape, it was possible to estimate the vertical displacements of protrusion tips by ignoring cell deformations and assuming an exponential relationship between spot brightness and distance between the cell tip and the substratum, according to Eq. (1). This analysis was expected to provide *an upper boundary to the amplitude of vertical cell fluctuation*.

The fluorescence profile of a typical spot is displayed on Fig. 2e: about 2/3 of total *specific* fluorescence (i.e., fluorescence calculated after background subtraction) is enclosed in a disc of 320 nm radius. The average brightness of a disc of this size centered on the spot center was considered as a robust reporter of total fluorescence. The specific brightness *I*_s_ was calculated by subtracting the background fluorescence from the average disc fluorescence. Following Eq. (1), we wrote:

3 $$ \ln \left( {I_{\text{s}} } \right) = A - z/d $$

Assuming that the major part of *I*_s_ variations was a consequence of the high dependence of fluorescence excitation on the distance *z* between the cell protrusion and the substratum, which amounted to neglecting the dependence on *z* of the fraction of fluorescence collected in the reference disc of 320 nm radius, the following relationship was obtained:

4 $$ {\text{SD}}\left( {\ln \left( {I_{\text{s}} } \right)} \right) \, = \, SD\left( z \right)/d $$

where SD stands for “standard deviation”.

Twenty-seven representative spots were monitored with a total number of 1808 positions (i.e., between 31 and 135 positions per spot). The mean SD of *I*_s_ was 6.5 ± 0.50 SEM, which is about twofold higher than the SD of 2.3 ± 0.50 SEM measured on an empty region with the same area. Using the well-known law of variance additivity, it appeared reasonable to estimate the intrinsic fluctuations of microvillus tip brightness as:

5 $$ {\text{SD}}_{i} = \left( {6.5^{2} - 2.3^{2} } \right)^{1/2} = 6.07 $$

Thus, measured brightness fluctuation should provide an approximate value of intrinsic brightness fluctuations of monitored objects. It was thus felt acceptable to use Eq. (4) to derive *z* fluctuations from brightness fluctuations.

Using the aforementioned determination^5^ of 0.0015 nm^−1^ for 1/d, the average vertical fluctuation of microvilli tips during a second was estimated at 67 nm ± 0.83 nm SEM, on the basis of 1703 sliding periods of 1 s, involving 5 brightness measurements each. When the duration of sliding periods was increased to 5 s, the calculated *z* fluctuation increase only by 50%, yielding 103 ± 1.03 nm SEM, thus suggesting that most fluctuations occurred within less than 1 s.

In order to obtain a more intuitive view of vertical cell motion, the fluorescence fluctuations of two representative spots simultaneously measured on a same cell are displayed in Fig. 2f. Results are consistent with the possibility that transverse motions of cell microvilli might appear as a combination of subsecond fluctuations of about 67 nm amplitude superimposed with slower motion of 5–10 s period and submicrometer amplitude.

#### Assessing the Potential Contribution of Brownian Motion to Estimated Fluctuations

**Figure 3:**
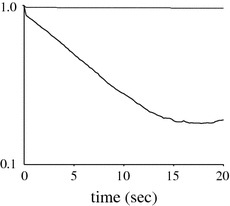
Dynamics of the T cell surface. The temporal autocorrelation function^12^ was determined on live and aldehyde-treated T lymphocytes after membrane labeling with Dia and observation of a 2 × 2 *µ*m^2^ area with TIRFM. Results obtained with a representative live (thick line) and fixed (thin line) are shown

It was important to determine the relative contribution of active cell movements and brownian motion in measured displacement of microvilli. This question was addressed by subjecting aldehyde-fixed cells to the same experimental study as live cells. Two important results were obtained. First, as expected, fixed cells displayed minimal deformation during observation periods of several tens of seconds. This is illustrated on Fig. 3: the temporal autocorrelation function of the image of a squared region of 2 × 2 *µ*m^2^ size remain nearly constant during a 20 s period in contrast to the temporal autocorrelation function of live cells. Second, fixed cells displayed much more significant brownian motion that live cells. About half of the studied spot-like microvilli tips displayed minimal lateral motion, as found on live cells, and the amplitude of fluctuations calculated on 1-s intervals was 24.0 nm ± 0.27 nm SEM (1562 measured values). Other spots displayed rapid motions that were highly suggestive of overall brownian fluctuations since all spots appearing on a same cells moved in parallel. The estimated fluctuation amplitude was 49.4 nm ± 0.73 nm SEM (1994 measured values).

These results strongly suggested that (i) weak nonspecific interactions should restrain the brownian motion of most live cells and a significant number of aldehyde-fixed cells touching IgG-coated surfaces, and (ii) active vertical movements of individual microvilli accounted for most of the brightness fluctuations measured on live cells. Indeed, using Eq. (5), the amplitude of active *z* fluctuation would be reduced from 67 nm to 62.5 nm after correcting for an estimated 24 nm contribution of brownian motion.

#### Assessing the Error Involved in Relating Brightness Fluctuations to Vertical Displacements

As indicated above, a number of approximations were involved in the use of Eqs. (3) and (4). Two points must be considered.

##### Diffraction Effects

**Figure 4:**
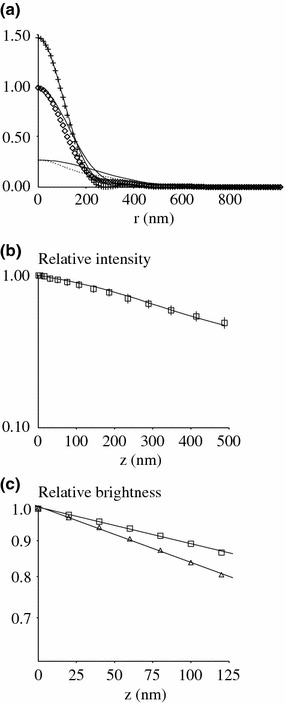
Relating pixel brigthness to z coordinate. (a) PSF of defocused fluorescent points. The PSFs of fluorescent points separated from the focus plane by a distance of 0 nm (crosses), 200 nm and 400 nm (points) were calculated with Gibson-Lanni’s model^13^ and numerical curves were approximated with Gaussian curves (thin lines) by fitting the maximum brighness and integrated density. (b) Simulating the TIRFM image of a fluorescent sphere. Squares represent the experimental radial dependence of relative brightness of the TIRM image of a fluorescent sphere of 5 *µ*m radius.^5^ Each value is the mean of ten images and verticar bar length is the SEM. This experimental curve is fitted by the following biexponential law: *I* = 0.56 exp(−0.0028z) + 0.44 exp(−0.00047z), where the distance *z* between the point and the focal plane is expressed in nm. The thin line is the simulated images built with numerical gaussian approximation of PSFs and TIRF illumination given by: *I* = 0.5 exp(−0.0015z) + 0.5. (c) Relationship between brightness and height of microvilli tips. Microvilli were modeled as cylinders of 160 nm radius and 320 nm (squares) or 480 nm (triangles) length cylinders born by a flat disc. The relationship between image brightnesses measured as described for cell images and distance to the coverslip are shown. Lines show the linear regression derived from calculated points on a logarithmic scale. Slopes are 0.00119 nm^−1^ (squares) and 0.00183 nm^−1^ (triangles)

As previously emphasized,^26^ the illumination intensity on defocused points is not exactly described by a single exponential, particularly when through-the-objective illumination is performed. Further, the image of a defocused fluorescent spot does not follow the standard Airy pattern. We used theoretical point spread functions^13^ approximated with gaussian functions by matching the maximum intensity and integrated density. As expected, and as illustrated on Fig. 4a, the fit between numerically obtained PSFs and gaussian functions appeared satisfactory when defocus *z* was lower than about 200 nm. While mismatch increased for higher *z* values, the error was estimated as acceptable due to the exponential decrease of total illumination intensity.

Interpolated Gaussian functions were used to build the image of a large sphere of 5 *µ*m radius and compare it to experimental data.^5^ The match between calculated and experimental values after numerical fitting of illumination intensity is shown on Fig. 4b.

##### Effect of Cell Shape

A bright disc is not a realistic model for a cell protrusion. We tried to account for the local geometry of the cell surface by modeling protrusions as cylinders with flat top of 160 nm radius arising from cells modeled as flat surfaces. This value was consistent with electron-microscopic data, and yielded fluorescent spots matching experimental images such as are shown on Fig. 1 the relationship between fluorescence intensity and coordinate *z* of microvilli tips is shown on Fig. 4c: the estimated decay constant (1/d) was respectively 0.0012 nm^−1^ and 0.00,183 nm^−1^ for a protrusion length of 320 nm and 480 nm.

Note that these values are consistent with both TIRFM images and electron microscopy of lymphocyte microvilli.^33^ Thus, Eq. (1) might be used as a first approximation, with an uncertainty of order of 20% on the decay constant that was tentatively taken as 0.0015 nm^−1^.

## Conclusion

The purpose of this paper was to obtain quantitative information on T-cell membrane dynamics at interfaces with subsecond and submicrometer resolution. Indeed, these phenomena are expected to play an important role in the T-cell capacity to probe its environment.^14^ Previous work done with IRM had revealed the occurrence of transverse undulations with a period of the order of seconds and *spatially averaged amplitude* of a few nanometers on surface areas of the order of several tenths of *µ*m^2^. Here, TIRFM was found to provide more accurate spatial information by revealing vertical displacements of individual microvilli with about 67 nm amplitude and subsecond frequency. Since cellular contacts involve membrane receptors of less than 10 nm length, membrane movements of submicrometer amplitude have the capacity to control ligand-receptor interactions. It must be emphasized that rapid displacements of subsecond frequency might strongly influence receptor binding properties: indeed it was shown with model systems that biomolecule interactions might be highly sensitive to variations of contact duration within the subsecond range.^24^ The following conclusions may be emphasized:

Our results are consistent with a number of recently reviewed reports^31^ suggesting that most cell membranes display continual fluctuations within the nanometer and second scale. Generated forces are likely to generate signaling cascades through mechanotranduction mechanisms that were evidenced in a number of cell types,^9^ including T lymphocytes.^20^,^22^ Membrane fluctuations might thus play a major role in environment probing by living cells.^31^

While the method we described is based on standard TIRFM images and may be easily applied to other cellular models, there are a number of approximations underlying our analysis. In addition to necessary approximations to the basic equations of optics, it had to be assumed that (i) nonspecific interactions between IgG-coated surfaces were weak enough to induce minimal perturbation on the movement of cell microvilli, (ii) cell deformations did not significantly contribute brightness variations on 1-s intervals, and (iii) while images such as are shown in Fig. 2 were not indicative of the presence of dye aggregates on the cell surface, it is difficult to exclude the possibility that dye heterogeneity might influence quantitative conclusions. Thus, more work is required to improve the accuracy of distance estimates.

In the present stage, our method might be used for semi-quantitative monitoring of the first step of T-lymphocyte activation by artificial surfaces. This might provide a new way of assessing lymphocyte function in medical practice as well as exploring the basic mechanism of T-lymphocyte activation.

## Abbreviations

APC: Antigen presenting cell
diA: 4-Di-16-ASP (4-(4-(dihexadecylamino)styryl)-*N*-methylpyridinium iodide)
IRM: Interference reflection microscopy
MHC: Major histocompatibility complex
*p*: Oligopeptide
PSF: Point spread function
RICM: Reflection interference contrast microscopy
SD: Standard deviation
SEM: Standard error of the mean
TCR: T cell receptor
TIRF: Total internal reflection fluorescence
TIRFM: Total internal reflection fluorescence microscopy

## References

[1] Ajo-Franklin CM, Ganesan PV, Boxer SG (2005). Variable incidence angle fluorescence interference contrast microscopy for z-imaging single objects. Biophys. J..

[2] Aleksic M, Dushek O, Zhang H, Shenderov E, Chen JL, Cerundolo V, Coombs D, van der Merwe PA (2010). Dependence of T cell antigen recognition on T cell receptor-peptide MHC confinement time. Immunity.

[3] Axelrod D (1981). Cell-substrate contacts illuminated by total internal reflection fluorescence. J. Cell Biol..

[4] Bongrand P, Malissen B (1998). Quantitative aspects of T-cell recognition: from within the antigen-presenting cell to within the T cell. Bioessays.

[5] Brodovitch A, Bongrand P, Pierres A (2013). T lymphocytes sense antigens within seconds and make a decision within one minute. J. Immunol..

[6] Bunnell SC, Kapoor V, Trible RP, Zhang W, Samelson LE (2001). Dynamic actin polymerization drives T cell receptor-induced spreading: a role for the signal transduction adaptor LAT. Immunity.

[7] Crétel, E., D. Touchard, A.-M. Benoliel, P. Bongrand, and A. Pierres. Early contacts between T-lymphocytes and activating surfaces. *J. Phys. Condens. Matter*. 22:194107, 2010. 10.1088/0953-8984/22/19/194107 21386434

[8] Crétel E, Touchard D, Bongrand P, Pierres A (2011). A new method for rapid detection of T lymphocyte decision to proliferate after encountering activating surfaces. J. Immunol. Methods.

[9] del Rio A, Perez-Jimenez R, Liu R, Roca-Cusachs P, Fernandez JM, Sheetz MP (2009). Stretching single talin rod molecules activates vinculin binding. Science.

[10] Evavold BD, Allen PM (1991). Separation of IL-4 production from Th cell proliferation by an altered T cell receptor ligand. Science.

[11] Feinerman O, Germain RN, Altan-Bonnet G (2008). Quantitative challenges in understanding ligand discrimination by αβ T cells. Mol. Immunol..

[12] Gaborski TR, Sealander MN, Ehrenberg M, Waugh RE, McGrath JL (2010). Image correlation microscopy for uniform illumination. J. Microsc..

[13] Gibson SF, Lanni F (1992). Experimental test of an analytical model of aberration in an oil-immersion objective lens used in three-dimensional light microscopy. J. Opt. Soc. Am..

[14] He H, Bongrand P (2012). Membrane dynamics shape TCR-generated signaling. Front. Immunol..

[15] Hiraoka Y, Sedat JW, Agard DA (1990). Determination of three-dimensional imaging properties of a light microscope system—partial confocal behavior in epifluorescence microscopy. Biophys. J..

[16] Hocdé SA, Hyrien O, Waugh RE (2009). Molecular accessibility in relation to cell topography and compression against a flat substrate. Biophys. J..

[17] Hocdé SA, Hyrien O, Waugh RE (2009). Cell adhesion molecule distribution relative to neutrophil surface topography assessed by TIRFM. Biophys. J..

[18] Huang JV, Zarnitsyna I, Liu B, Edwards LJ, Jiang N, Evavold BD, Zhu C (2010). The kinetics of two-dimensional TCR and pMHC interactions determine T-cell responsiveness. Nature.

[19] Huppa JB, Axmann M, Mörtelmaier MA, Lillemeier BF, Newell EW, Brameshuber M, Klein LO, Schütz GJ, Davis MM (2010). TCR-peptide-MHC interactions in situ show accelerated kinetics and increased affinity. Nature.

[20] Kim, S. T., K. Takeuchi, Z.-Y. J. Sun, M. Touma, C. E. Castro, A. Fahmy, M. J. Lang, G. Wagner, and E. L. Reinherz. The αβ T cell receptor is an anisotropic mechanosensor. *J. Biol. Chem*. 284:31028–31037, 2009. 10.1074/jbc.M109.052712 PMC2781503 19755427

[21] Kirshner H, Aguet F, Sage D, Unser M (2013). 3D-PSF fitting for fluorescence microscopy: implementation and localization application. J. Microsc..

[22] Li, Y.-C., B.-M. Chen, P.-C. Wu, T.-L. Cheng, L.-S. Kao, M.-H. Tao, A. Lieber, and S.R. Roffler. Cutting edge: mechanical forces acting on T cells immobilized via the TCR complex can trigger TCR signaling. *J. Immunol*. 184:5959–5963, 2010. 10.4049/jimmunol.0900775 20435924

[23] Liu B, Chen W, Evavold BD, Zhu C (2014). Accumulation of dynamic catch bonds between TCR and agonist peptide-MHC triggers T cell signaling. Cell.

[24] Lo Schiavo V, Robert P, Limozin L, Bongrand P (2012). Quantitative modeling assesses the contribution of bond strengthening, rebinding and force sharing to the avidity of biompolecule interactions. PLoS ONE.

[25] Matsui K, Boniface JJ, Steffner P, Reay PA, Davis MM (1994). Kinetics of T-cell receptor binding to peptide/I-Ik complexes: correlation of the dissociation rate with T-cell responsiveness. Proc. Natl Acad. Sci. USA.

[26] Mattheyses AL, Axelrod D (2006). Direct measurement of the evanescent field profile produced by objective-based total internal reflection fluorescence. J. Biomed. Optics.

[27] Mattheyses AL, Simon SM, Rappoport JZ (2010). Imaging with total internal reflection fluorescence microscopy for the cell biologist. J. Cell Sci..

[28] Paszek MJ, DuFort CC, Rubashkin MG, Davidson MW, Thorn KS, Liphardt JT, Weaver VM (2012). Scanning angle interference microscopy reveals cell dynamics at the nanoscale. Nat. Methods.

[29] Pierres, A., P. Eymeric, E. Baloche, D. Touchard, A.-M. Benoliel, and P. Bongrand. Cell membrane alignment along adhesive surfaces: contribution of active and passive cell processes. *Biophys. J*. 84:2058–2070, 2003. 10.1016/S0006-3495(03)75013-9 PMC1302774 12609907

[30] Pierres A, Benoliel A-M, Touchard D, Bongrand P (2008). How cells tiptoe on adhesive surfaces before sticking. Biophys. J..

[31] Pierres A, Monnet-Corti V, Benoliel A-M, Bongrand P (2009). Do membrane undulations help cells probe the world?. Trends Cell Biol..

[32] Robert P, Aleksic M, Dushek O, Cerundolo V, Bongrand P, van der Merwe PA (2012). Kinetics and mechanics of 2D interactions between T cell receptors and different activating ligands. Biophys. J..

[33] Sage PT, Varghese LM, Martinelli R, Sciuto TE, Kamei M, Dvorak AM, Springer TA, Sharpe AH, Carman CV (2012). Antigen recognition is facilitated by invadosome-like protrusions formed by memory/effector T cells. J. Immunol..

